# An anthropometric evidence against the use of age-based estimation of bodyweight in pediatric patients admitted to intensive care units

**DOI:** 10.1038/s41598-023-30566-3

**Published:** 2023-03-02

**Authors:** Nobuyuki Nosaka, Tatsuhiko Anzai, Ryo Uchimido, Yuka Mishima, Kunihiko Takahashi, Kenji Wakabayashi

**Affiliations:** 1 Department of Intensive Care Medicine, Graduate School of Medical and Dental Sciences, Tokyo Medical and Dental University, 1-5-45 Yushima, Bunkyo-Ku, Tokyo, 113-8510 Japan; 2 Department of Biostatistics, M&D Data Science Center, Tokyo Medical and Dental University, Tokyo, Japan

**Keywords:** Paediatric research, Physical examination, Body mass index

## Abstract

Age-based bodyweight estimation is commonly used in pediatric settings, but pediatric ICU patients often have preexisting comorbidity and resulting failure to thrive, hence their anthropometric measures may be small-for-age. Accordingly, age-based methods could overestimate bodyweight in such settings, resulting in iatrogenic complications. We performed a retrospective cohort study using pediatric data (aged < 16 years) registered in the Japanese Intensive Care Patient Database from April 2015 to March 2020. All the anthropometric data were overlaid on the growth charts. The estimation accuracy of 4 age-based and 2 height-based bodyweight estimations was evaluated by the Bland–Altman plot analysis and the proportion of estimates within 10% of the measured weight (ρ10%). We analyzed 6616 records. The distributions of both bodyweight and height were drifted to the lower values throughout the childhood while the distribution of BMI was similar to the general healthy children. The accuracy in bodyweight estimation with age-based formulae was inferior to that with height-based methods. These data demonstrated that the pediatric patients in the Japanese ICU were proportionally small-for-age, suggesting a special risk of using the conventional age-based estimation but supporting the use of height-based estimation of the bodyweight in the pediatric ICU.

## Introduction

Anthropometric measurements (e.g. bodyweight, height, and head circumferences) are important to determine the dosage of medications and the equipment size for pediatric patients^1^. These anthropometric indices undergo dramatic changes as children grow during their entire childhood^2^, and numerous age-based and height-based methods have been proposed to guide appropriate medical interventions. These estimation methods are particularly important in pediatric emergency and intensive care settings, where immediate medical interventions are often required before measuring bodyweight on site, thus various age-based estimation formulas for estimating bodyweight^3–7^ have been proposed because precise age information is readily available in most cases.

However, it is notable that these age-based estimation formulae were developed based on general populations^5–10^, and pediatric patients admitted to intensive care units (ICUs) may not follow the anthropometric archetype of the general population because pediatric patients in the ICU often have preexisting comorbidity and resulting failure to thrive^11–13^. A couple of studies have provided anthropometric characteristics of the pediatric population admitted to ICUs. In a prospective British single-center study^14^, the pediatric population in the ICU had significantly lower weight-for-age compared to the general British children, with the increased proportion of extremely low weight-for-age (18% of the study population were less than − 2.5 SD below the UK reference population mean bodyweight). Ross et al.^15^, using a large retrospective analysis of prospectively collected data from multiple pediatric ICUs in the United States, also showed that pediatric ICU patients had lower weight-for-age compared to the general US population. From the perspective of medical safety, this evidence collectively implicated that the use of age-based estimation of anthropometric values may pose a risk to the pediatric ICU population because of drug dosage errors^5^. However, the performance comparison of different bodyweight estimation methods has not yet been well explored for the pediatric ICU population.

In this study, we aimed to characterize anthropometric data of the pediatric ICU population in Japan, and evaluate the validity of age-based bodyweight estimation methods for the pediatric ICU population. We hypothesized that the pediatric population in ICU is proportionally small-for-age, hence height-based bodyweight estimation should be used for the pediatric patients in the ICU.

## Methods

In this study, we aimed to investigate the distribution of anthropometric indices (bodyweight, height, and body mass index [BMI, identical to the Kaup index]) of Japanese children in ICU on the growth charts. We also aimed to evaluate the performance of established age-based bodyweight calculation tools compared with height-based estimation methods for the pediatric population in Japanese ICU.

### Study design and cohort

We performed a retrospective cohort study using the data derived from the Japanese Intensive Care Patient Database (JIPAD), a national intensive care unit registry in Japan^16^. We obtained the 5-year JIPAD data of consecutive patients aged less than 16 years who were admitted to ICU from April 2015 to March 2020. The database provides patient demographics and anthropometric data including bodyweight and height^16^. This study was reviewed and approved, and the need for informed consent was waived considering the retrospective design and complete anonymization, by Tokyo Medical and Dental University Review Board (M2020-245). All methods in our study were performed in accordance with the relevant guidelines and regulations.

### Data plotting on growth charts and standard deviation score calculation

All height and bodyweight data were plotted on the growth charts for Japanese children^17^ officially provided online by the Japanese Society for Pediatric Endocrinology (JSPE; http://jspe.umin.jp/medical/chart_dl.html, Accessed on April 2021). Percentile data of bodyweight and height for each age were calculated by using R software, version 4.1.2 (The R Foundation for Statistical Computing, Vienna, Austria)*.*

To quantitatively compare the anthropometric indices of pediatric ICU patients with the above JSPE reference-standard, we used standard deviation scores (SDS) for bodyweight, height, and BMI as previously described^15,18,19^. The SDS for each anthropometric index was calculated using the Excel-based Clinical Tool for Growth Evaluation of Children provided by the JSPE (A general version can be downloaded at http://jspe.umin.jp/medical/chart_dl.html, Accessed on April 2021. A special version for big data analysis was kindly provided by Dr. Yoshiya Ito on behalf of JSPE). Each index required age-in-month to calculate, although the JIPAD database provides age-in-year for subjects aged more than three years. Therefore, for subjects aged three years or older, we calculated these indices using a surrogate age-in-month of “12 × (age) + 6” (e.g. 126 months-old for 10-year-old subjects). Patients were classified into the “extremely low” category for each index when the index was less than − 2.5 SD of the general Japanese population mean^14^.

Statistical analysis for the distribution of anthropometric data was performed using PRISM 7 (GraphPad) and R software (The R Foundation for Statistical Computing).

### Validity assessment of bodyweight estimation tools

We evaluated the validity of a total of six bodyweight estimation methods (Supplementary material 1): four age-based formulae (the original APLS formula^20^, the new APLS formula^21^, the Best Guess formula^10^, and the JAPAN formulae^5^) and two height-based methods (Broselow Pediatric Emergency Reference Tape 2019 edition [BT^22^; Vyaire Medical, Inc., Mettawa, IL, USA], and the JAPAN scale^23^). We chose the above 6 methods because we have recently developed and validated the age-based JAPAN formulae and the height-based JAPAN scale for bodyweight estimation for children using a Japanese large nationwide longitudinal survey^5,23^, and the other selected formulae have been commonly applied for bodyweight estimation and widely evaluated internationally^3^ although the covered age range varies according to the formulae (Supplementary material 1). Instead of fitting Broselow “Tape” to the actual patients, height data were cross-referenced to the BT scale and the JAPAN scale upon height-based bodyweight estimation. Notably, the covered height range varies according to the scales (Supplementary material 1).

The Bland–Altman approach and the proportions of the estimates within 10% of the recorded weight (ρ10%) were used to evaluate the accuracy and precision of the estimation methods as previously described^3,4,24,25^. We generated Bland–Altman plots to visually evaluate the agreement between the recorded and estimated bodyweight and calculated the bias and 95% limits of agreement (LOA)^26^. The resulting graph describes the difference of the two values (recorded and estimated bodyweight) plotted (the Y axis) against the mean of the two values (the X axis). The bias represents the difference between the recorded and estimated bodyweight where positive and negative values indicate under- and over-estimation of the bodyweight on average, respectively. The 95% LOA shows the interval in which 95% of the differences between the recorded and estimated bodyweight will fall.

While smaller bias and narrow 95% LOA interval mean a better estimation method, the ρ10% should be as large as possible to be a reliable bodyweight estimation method^3,4,24,25^. In this study, we also assessed ρ15% and ρ20% to reinforce the findings. In addition, we also evaluated the proportions of estimates within absolute difference (2 kg and 4 kg) of the recorded weight, because the percentage difference would carry different impacts depending on the recorded weight in pediatric patients (e.g. The 10% difference for a 10-kg child is 1 kg while it becomes 5 kg for a 50-kg child).

### Ethical approval and consent to participate

The study was approved by the Tokyo Medical and Dental University Review Board (M2020-245) as well as the steering committee of JIPAD, and anonymized data were provided for analysis by the JIPAD.

## Results

### Pediatric ICU patients are proportionally small

**Figure 1:**
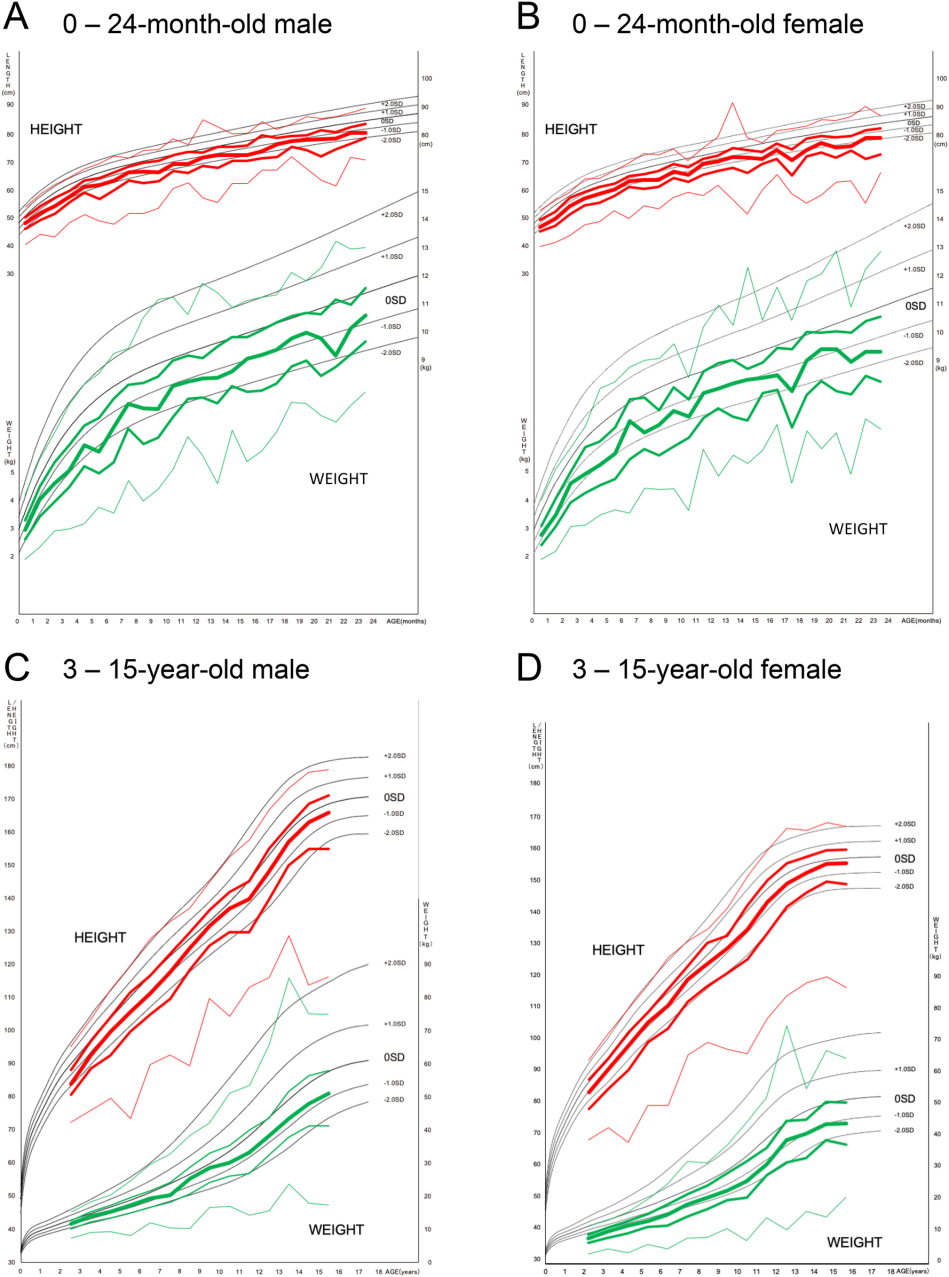
Percentile distribution of height and bodyweight of pediatric patients admitted to intensive care unit. Percentile distribution of height (red lines) and bodyweight (green lines) were overdrawn on the growth charts for Japanese children (black lines, reference #2. The charts were reproduced with official permission of the Japanese Society for Pediatric Endocrinology from Isojima et al. Growth standard charts for Japanese children with mean and standard deviation (SD) values based on the year 2000 national survey. *Clin Pediatr Endocrinol* 25: 71–76, 2016. ©JSPE). The 1st (fine), 2nd (middle), 3rd (bold), 4th (middle), and 5th (fine) lines from the top indicate 97.5 percentile, 75.0 percentile, 50 percentile, 25 percentile and 2.5 percentile, respectively. Crude data is shown in Supplementary material 2.

**Figure 2:**
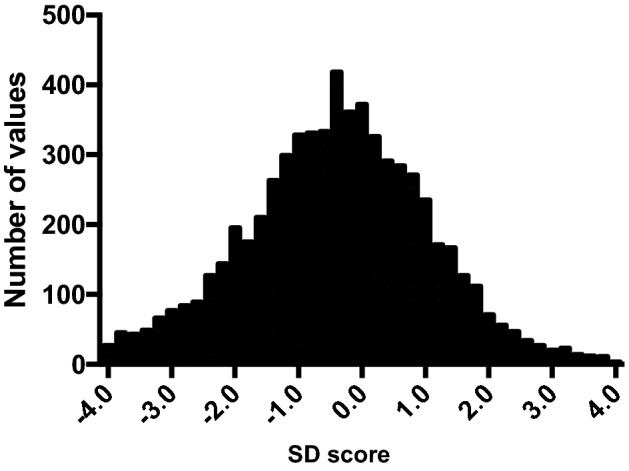
Distributions of SD score of BMI of pediatric population in intensive care units. The bin width of each bar is 0.2. There are 278 data points outside the axis limit. SD, standard deviation; BMI, body mass index.

A total of 7433 admission records from 60 facilities in the JIPAD database were identified in the study period: We excluded 113 records due to missing or improbable data and 704 readmissions within the same hospital stay. We analyzed 6616 admission records with complete data for age, sex, height, and bodyweight. The characteristics of the overall study cohort was presented in Table 1. Overall, the distributions of both bodyweight and height were shifted to the lower side (Fig. 1; the detailed data were shown in the Supplementary material 2 and 3) with approximate mean SDS of − 1.2 and around 20% of patients categorized in the extremely low category (Table 1). The distribution of BMI is almost bell-shaped (Fig. 2) and had higher mean SDS of − 0.52 (95% CI − 0.57 to − 0.48) with less subjects in the extremely low category (10.7%, Table 1). The disease category subgroup analysis revealed that subjects admitted due to “cardiovascular”, “respiratory”, or “gastrointestinal” diseases had lower mean SDSs than the other categories, mainly contributing to expansion of the population in extremely low categories (Table 2).

**Table 1:** Characteristics of study population.

N	6616
Age (year), median [IQR]	2.0 [0.0, 8.0]
Female [numbers (%)]	3029 (45.8)
Elective admission [numbers (%)]	4184 (63.2)
Post-operation [numbers (%)]	4653 (70.3)
Bodyweight SDS [mean (SD)]	− 1.27 (2.36)
Bodyweight category [numbers (%)]	
SDS < − 2.5	1308 (19.8)
− 2.5 ≦ SDS < 2.5	5266 (79.6)
SDS ≧ 2.5	42 (0.6)
Height SDS [mean (SD)]	− 1.21 (2.13)
Height category [numbers (%)]	
SDS < − 2.5	1391 (21.0)
− 2.5 ≦ SDS < 2.5	5138 (77.7)
SDS ≧ 2.5	87 (1.3)
BMI SDS [mean (SD)]	− 0.52 (1.91)
BMI category [numbers (%)]	
SDS < − 2.5	706 (10.7)
− 2.5 ≦ SDS < 2.5	5714 (86.4)
SDS ≧ 2.5	196 (3.0)

BMI, body mass index; IQR, interquartile range; SD(S), standard deviation (score).

Patients with SDS < − 2.5 were classified into the “extremely low” category for each index.

**Table 2:** Characteristics of study population including details of distribution across demographic and anthropometrical indices.

N	Cardiovascular*		Respiratory		Neurological		Gastrointestinal		Trauma		Others**	
	1841		1539		1423		786		134		893	
Age (year), median [IQR]	0.0	[0.0, 3.0]	3.0	[1.0, 8.0]	4.0	[1.0, 9.0]	2.0	[0.0, 6.0]	6.5	[1.0, 10.0]	7.0	[2.0, 13.0]
Female [numbers (%)]	877	(47.6)	595	(38.7)	661	(46.5)	383	(48.7)	48	(35.8)	465	(52.1)
Elective admission [numbers (%)]	1509	(82.0)	822	(53.4)	791	(55.6)	539	(68.6)	0	(0.0)	523	(58.6)
Post-operation [numbers (%)]	1524	(82.8)	862	(56.0)	962	(67.6)	689	(87.7)	0	(0.0)	616	(69.0)
SDS [mean (SD)]												
Bodyweight	− 1.51	(2.12)	− 1.56	(2.83)	− 0.57	(1.77)	− 1.58	(2.67)	− 0.37	(1.34)	− 1.26	(2.29)
Height	− 1.30	(1.88)	− 1.47	(2.54)	− 0.74	(1.94)	− 1.58	(2.22)	− 0.16	(1.88)	− 1.14	(1.94)
BMI	− 0.84	(1.67)	− 0.53	(2.16)	− 0.04	(1.63)	− 0.58	(2.30)	− 0.34	(1.49)	− 0.61	(1.85)
Extremely low category (SDS < − 2.5) [numbers (%)]												
Bodyweight	422	(22.9)	362	(23.5)	149	(10.5)	193	(24.6)	3	(2.2)	179	(20.0)
Height	393	(21.3)	411	(26.7)	181	(12.7)	225	(28.6)	8	(6.0)	173	(19.4)
BMI	246	(13.4)	187	(12.2)	71	(5.0)	89	(11.3)	8	(6.0)	105	(11.8)

BMI, body mass index; IQR, interquartile range; SD(S), standard deviation (score).

*Disease category “Cardiovascular” diseases includes “Post-cardiopulmonary resuscitation”.

**Disease category “Others” includes “Sepsis”.

### The accuracy of age-based bodyweight estimation for pediatric ICU patients is low

**Figure 3:**
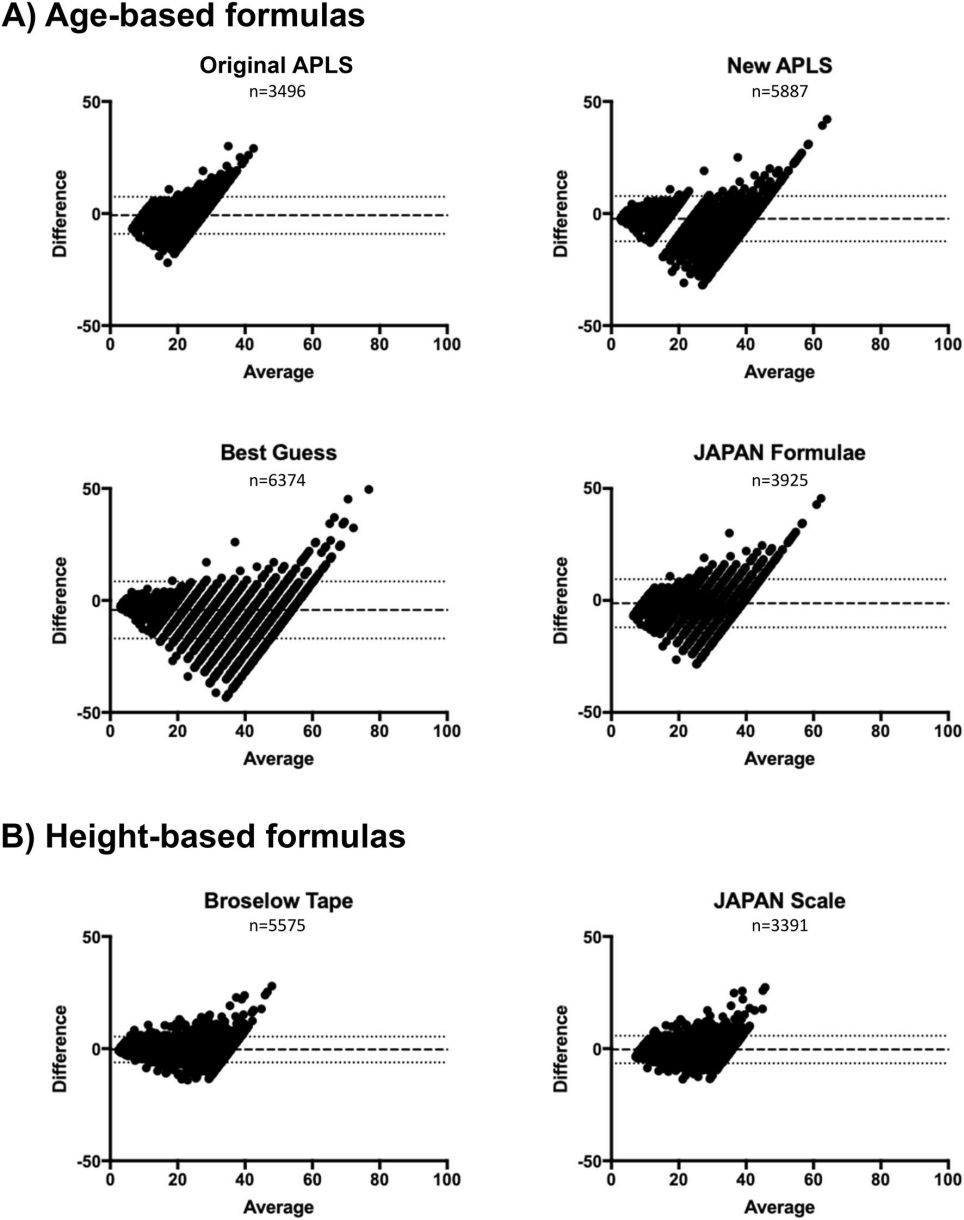
Bland–Altman plots for estimated bodyweight and measured bodyweight. Bland–Altman plots were drawn for 4 age-based formulas (**A**) and 2 height-based formulas (**B**). For the Bland–Altman plots, the long-dashed line indicates the bias, and the area between the short-dashed lines denotes the 95% limits of agreement. There is one data point plotted outside the axis limit in New APLS, Best Guess, JAPAN Formulae, and Broselow Tape, respectively.

The performance of the bodyweight estimation methods was visually summarized in Bland–Altman plots (BA-plots; Fig. 3). The BA-plots for the four age-based methods were more widely distributed than those of the height-based formulae. The BA analysis also provides quantitative assessment of the performance where the best estimation formula should give small bias and narrow 95% LOA interval. The bias of age-based formulae was farther to zero with the wider 95% LOA when compared with that of height-based methods, which indicated that age-based formulae had lower accuracy and precision than height-based methods. We also calculated the ρ10% which should be a larger value when the estimation formula performs better, and the overall accuracy of age-based formulae indicated by ρ10% was lower, compared to the two height-based methods (Table 3).

**Table 3:** Accuracy of each bodyweight estimation formula.

	Age-based formula				Height-based formula	
	Original APLS	New APLS	Best Guess	JAPAN Formulae	Broselow Tape	JAPAN Scale
Assessed number*	3496	5887	6374	3925	5575	3391
Values of Bland–Altman analysis						
Bias	− 0.8285	− 2.365	− 4.286	− 1.312	− 0.4335	− 0.4270
SD of Bias	4.222	5.147	6.498	5.486	2.933	3.123
95% LOA	− 9.103–7.446	− 12.45–7.723	− 17.02–8.450	− 12.06–9.440	− 6.183–5.316	− 6.548–5.694
Proportions of estimates						
ρ10% (%, 95%CI)	38.4 (36.8–40.1)	30.7 (29.5–31.9)	18.5 (17.6–19.5)	38.2 (36.7–39.8)	49.3 (48.0–50.6)	53.8 (52.1–55.5)
ρ15% (%, 95%CI)	52.7 (51.0–54.3)	43.4 (42.2–44.7)	28.1 (27.0–29.2)	51.6 (50.0–53.1)	66.5 (65.2–67.7)	70.6 (69.0–72.1)
ρ20% (%, 95%CI)	64.9 (63.3–66.5)	53.9 (52.6–55.2)	37.6 (36.4–38.8)	63.4 (61.8–64.9)	79.1 (78.0–80.1)	82.5 (81.2–83.7)
ρ2kg (%, 95%CI)	52.5 (50.8–54.1)	57.7 (56.5–59.0)	37.7 (36.5–38.9)	48.3 (46.8–49.9)	76.6 (75.5–77.7)	68.0 (66.4–69.6)
ρ4kg (%, 95%CI)	77.8 (76.4–79.2)	75.9 (74.8–77.0)	62.8 (61.6–64.0)	72.5 (71.1–73.9)	91.0 (90.3–91.8)	87.9 (86.8–89.0)

LOA; limits of agreement; SD: standard deviation;

ρ: proportion of estimates within indicated fraction of the measured weight, or within indicated bodyweight range; CI: confidence interval.

*The assessed numbers are different depending on the formulae because the covered age/height range varies (see Supplemental material 1).

## Discussion

Rapid and precise estimation of anthropometric values of children is important in an emergency room and ICU because they are key determinants for drug dosage and size of equipment. Several age-based bodyweight estimation formulae have been proposed because age is the most readily available information hence allowing us to immediately work out the answers even in urgent settings such as cardiopulmonary resuscitation, however, the accuracy of these formulae has been questioned^4^. Height-based estimation formulae such as Broselow Tape are also widely used methods, but their accuracy has also been challenged^3^. Importantly, these estimation methods were derived from general pediatric populations, therefore whether it is applicable to critically ill children has not been well elucidated. This is particularly important in the pediatric ICU where significant proportions of patients have preexisting comorbidities and resulting failure to thrive. In this study, we demonstrated detailed visual data on anthropometric characteristics of the pediatric ICU population in Japan where both the ICU database and the national pediatric anthropometric references have been long established^17^.

We have demonstrated that the distributions of bodyweight and height of pediatric ICU population are shifted to the lower side, in line with the previous studies^14,15^. We have also demonstrated that the proportion of extremely low weight-for-age/height-for-age reaches to around 20% of pediatric patients in ICU while the general prevalence of childhood stunting in developed countries including Japan is around 6%^27^. On the other hand, we have also described that BMI-for-age had a balanced bell-shaped distribution, which suggests that the body shape is maintained conformable to bodyweight and height for each age in this population. Considering that BMI is a practical assessment index for nutritional status^19,28^, we speculate that the possible major explanation for the distribution dissociation between weight-/height-for-age and BMI-for-age is the patients’ morbidity rather than the poor nutritional status. Indeed, most disease groups had lower SDS of weight- or height-for-age than that of BMI-for-age, whereas the subjects with “trauma”, which is an acutely acquired condition, had comparable values of these indexes which were closer to zero. In response, most disease group have more subjects in the extremely low categories of weight- or height-for-age than that of BMI-for-age, whereas few subjects with “trauma” belonged to the extremely low categories of these indexes (Table 2).

These “proportionally small-for-age” anthropometric characteristics explain why the height-based bodyweight estimation methods had superior validity over age-based methods for children in ICU. In line with this, Flannigan et al. have described that the age-based new APLS formulae can overestimate the bodyweight of PICU patients in the UK^29^ by approximately 20%. Moreover, as shown in the Supplementary material 3, the distribution in bodyweight has a wide range in each age, suggesting that mean-for-age bodyweight alone carries a high risk of misestimation of actual bodyweight. This evidence collectively agrees with the recent SCCM guideline for safe medication in ICU where BT was recommended to reduce medication errors^30^.

Importantly, the “proportionally small-for-age” anthropometric characteristics of pediatric ICU population could influence the safety upon device size selection; i.e. age-based methods could overestimate device size in this population, contributing to undesirable outcomes. For example, overestimating endotracheal tube size does matter for pediatric ICU population because this may result in multiple unrequired attempts at intubation and upper airway injury due to excessive pressures on the mucosa, leading to post-extubation sore throat^31,32^, or subglottic stenosis at worst^33,34^. Indeed, there are several studies which demonstrated the inferior ability of the age-based device size estimation over the other approaches^35–39^. Therefore, from the viewpoint of patient safety, we suggest avoiding the age-based device size estimation and choose alternative way (e.g. height-based estimation) given the pediatric ICU population has such “proportionally small-for-age” anthropometric trends.

This study was inherently subject to some limitations. First, we calculated SDS for subjects aged 3 years or older using surrogate age of “12 × (age) + 6” as explained in the Methods section. We performed the sensitivity analysis, for confirmation, with the SDS calculated using the most conservative surrogate age of “12 × (age)” for these subjects, in which the results produced the same conclusion as the original (data not shown). However, we did not perform the analysis with the SDS calculated using the surrogate age of “12 × (age) + 11” because it was evident that the calculated SDS becomes smaller as the reference age gets older. Second, similar to other databases^14,40^, the JIPAD database allows guardian-reported or estimated values in case measured values of bodyweight and height are not available. However, we have reported that the accuracy of mother-reported anthropometric values are extremely high in Japan (ρ10%: 94.9%, ρ20%: 98.7%)^24^. Third, our data confirmed the superiority of height-based methods over age-based methods for bodyweight estimation of pediatric ICU patients, however, the ρ10% of height-based methods in this study were still lower than those reported previously^4,41^. Accordingly, we recommend avoiding age-based methods, and using height-based methods until obtaining patients’ actual bodyweight information in these population.

## Conclusion

We demonstrated that the distributions of bodyweight and height of pediatric population in intensive care units are skewed toward small-for-age using prospectively collected database from 60 ICUs in Japan. Our results suggest a special risk of using age-based methods, and support relative but clear advantages of using height-based methods for patient safety, especially in pediatric ICU settings.

## Supplementary Information

Supplementary Information.

## Data Availability

The data that support the findings of this study are available from JIPAD, but restrictions apply to the availability of these data, which were used under permission for the current study, and are thus not publicly available. Data are however available from the authors upon reasonable request and with permission of the steering committee of JIPAD.

## Abbreviations

APLS: Advanced pediatric life support
BMI: Body mass index
BT: The Broselow tape
ICU: Intensive care units
IQR: Interquartile range
JIPAD: The Japanese Intensive Care Patient Database
JSPE: The Japanese Society for Pediatric Endocrinology
LOA: Limits of agreement
SD(S): Standard deviation (scores)
UK: The United Kingdom
US: The United States
