# Supplementary material for: An anthropometric evidence against the use of age-based estimation of bodyweight in pediatric patients admitted to intensive care units
Source: Sci Rep. 2023 Mar 2;13:3574. doi: 10.1038/s41598-023-30566-3 (PMC9981604; doi:10.1038/s41598-023-30566-3)
Supplement: Supplementary file 1 — Supplementary Information. [file 41598_2023_30566_MOESM1_ESM.pdf]

Supplementary Information

An anthropometric evidence against the use of age-based estimation of bodyweight in pediatric patients admitted to intensive care units

Nobuyuki Nosaka[1*], Tatsuhiko Anzai[2], Ryo Uchimido[1], Yuka Mishima[1], Kunihiko Takahashi[2], and Kenji Wakabayashi[1]

[1]Department of Intensive Care Medicine, Graduate School of Medical and Dental Sciences, Tokyo Medical and Dental University, Tokyo, Japan

[2]Department of Biostatistics, M&D Data Science Center, Tokyo Medical and Dental University, Tokyo, Japan

Supplementary material 1. Bodyweight Estimation Methods in this Study

Name	Formula or method	Covered range
Old APLS formula	(2×Age) + 8	Age 1 to 10
New APLS formula	(Month age/2) + 4	Age < 1
	(2×Age) + 8	Age 1 to 5
	(3×Age) + 7	Age 6 to 12
Best Guess formula	(2×Age) + 10	Age 1 to 5
	4×Age	Age 6 to 14
JAPAN formulae	(2×Age) + 9	Age 1 to 6
	(7×Age −3)/2	Age 7 to 12
BT 2019 edition	Total body weight estimation scale based on NHANES dataset.	Length 46.9 to 143.6cm
JAPAN scale	Total body weight estimation scale made for Japanese children.	Age 1 to 12. Length 72 to 144 cm.

NHANES, National Health and Nutrition Examination Survey

Supplementary material 2a.

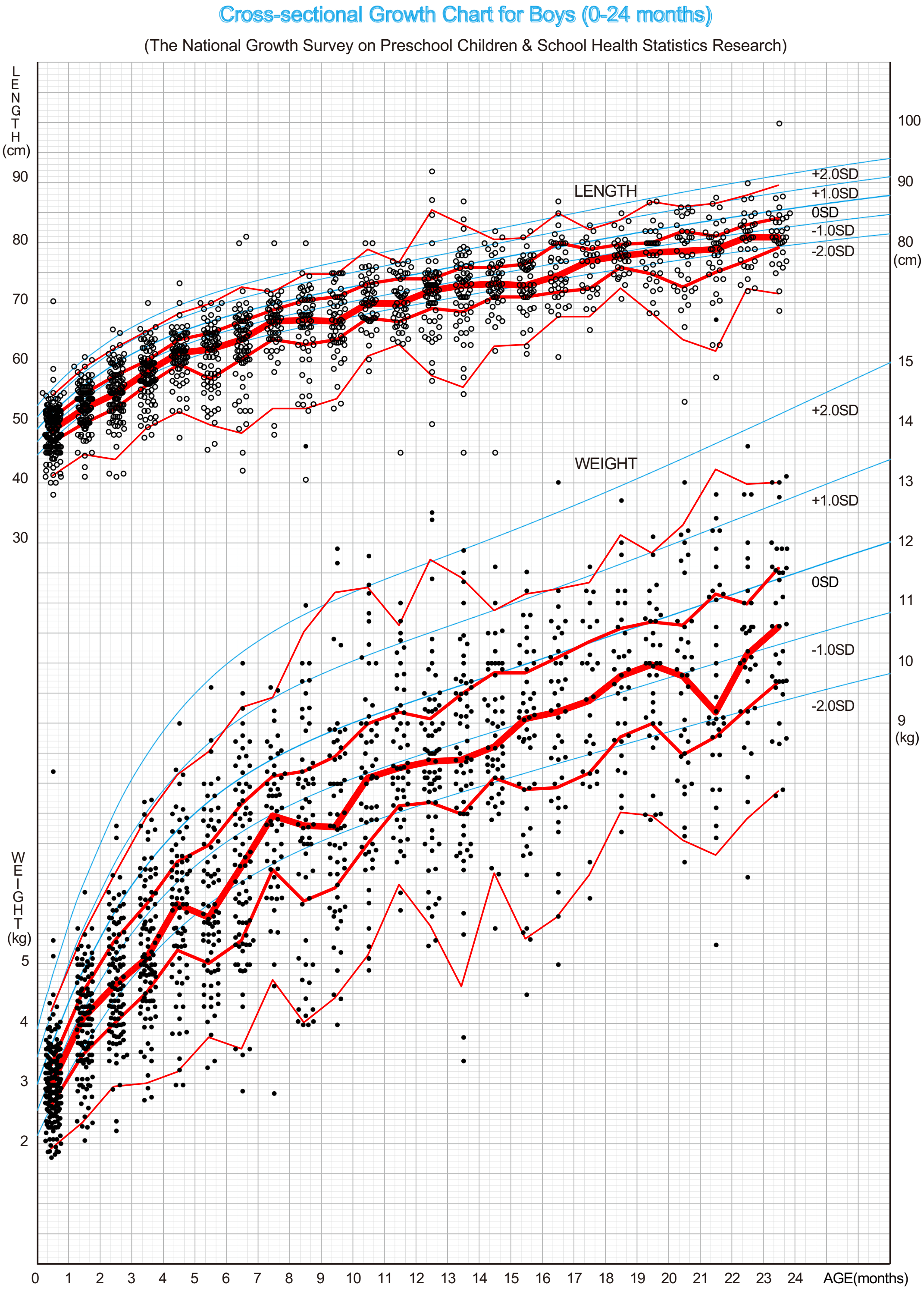

©The Japanese Society for Pediatric Endocrinology

Sources: (Length·Weight) N Kato, T Isojima, M Murata, et al: Clin Pediatr Endocrinol 25:71-76, 2016

Supplementary material 2b.

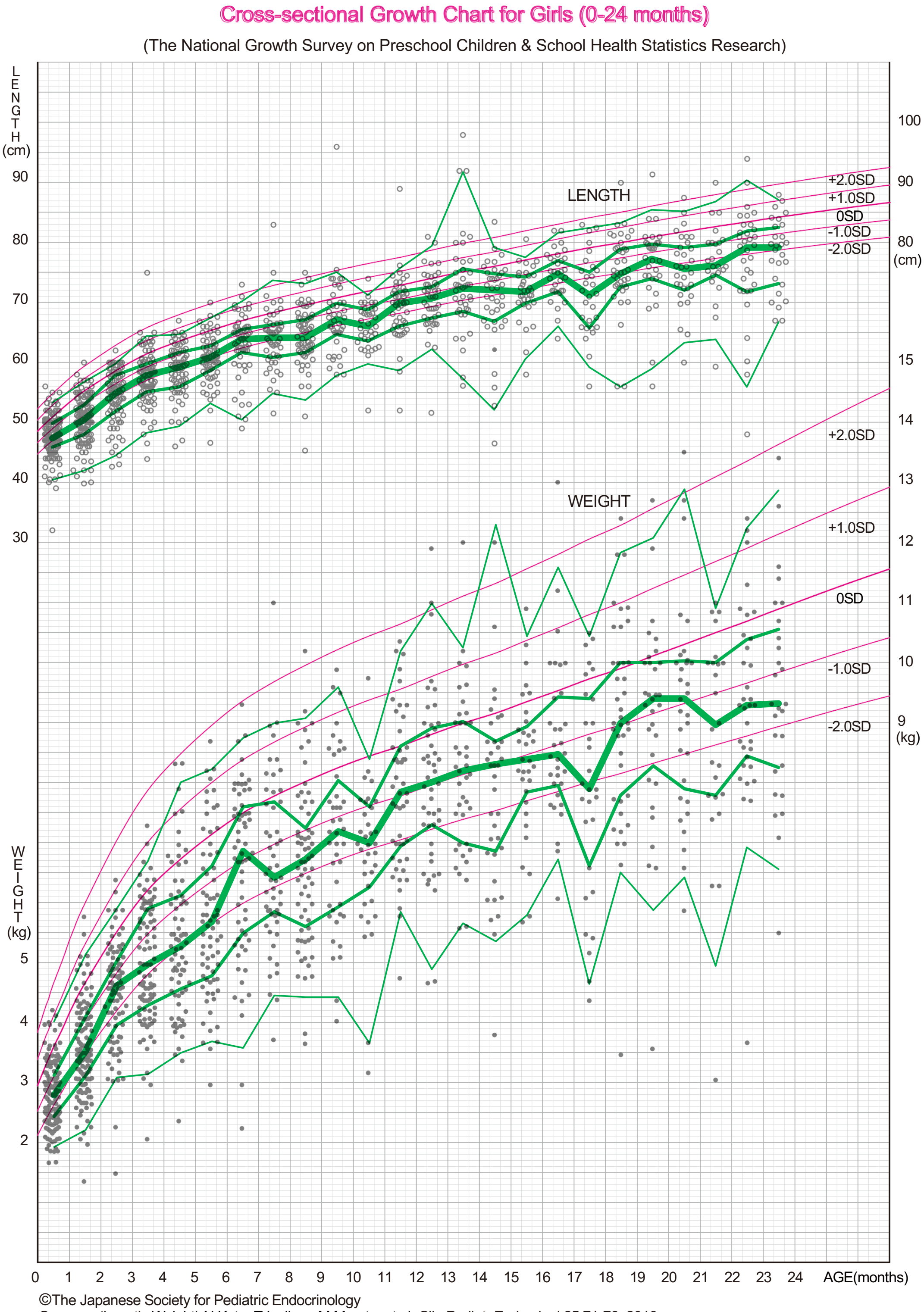

©The Japanese Society for Pediatric Endocrinology

Sources: (Length·Weight) N Kato, T Isojima, M Murata, et al: Clin Pediatr Endocrinol 25:71-76, 2016

Supplementary material 2c.

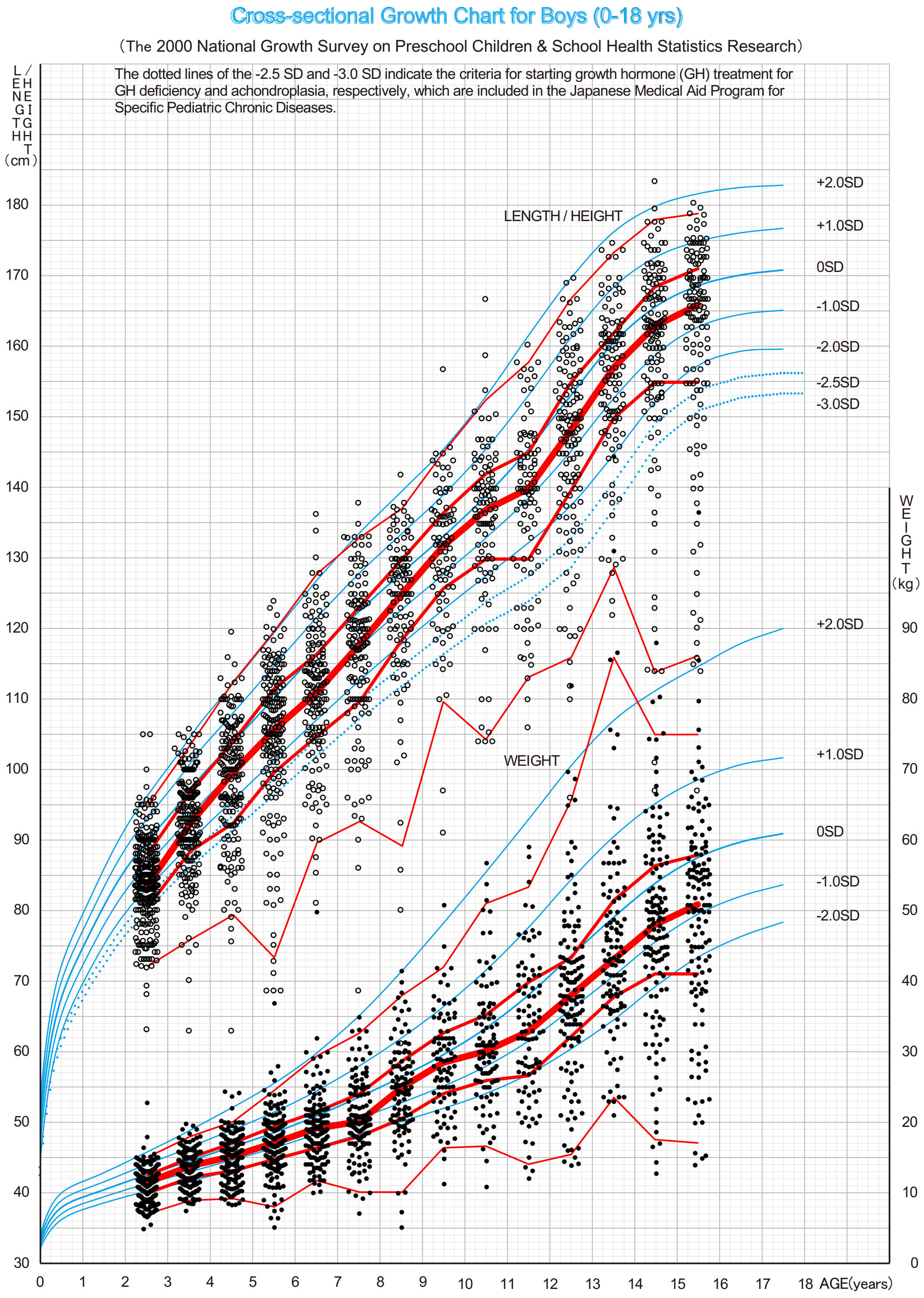

© The Japanese Society for Pediatric Endocrinology Source: T Isojima, N Kato, Y Ito, S Kanzaki, M Murata, Clin Pediatr Endocrinol 25:71-76, 2016

Supplementary material 2d.

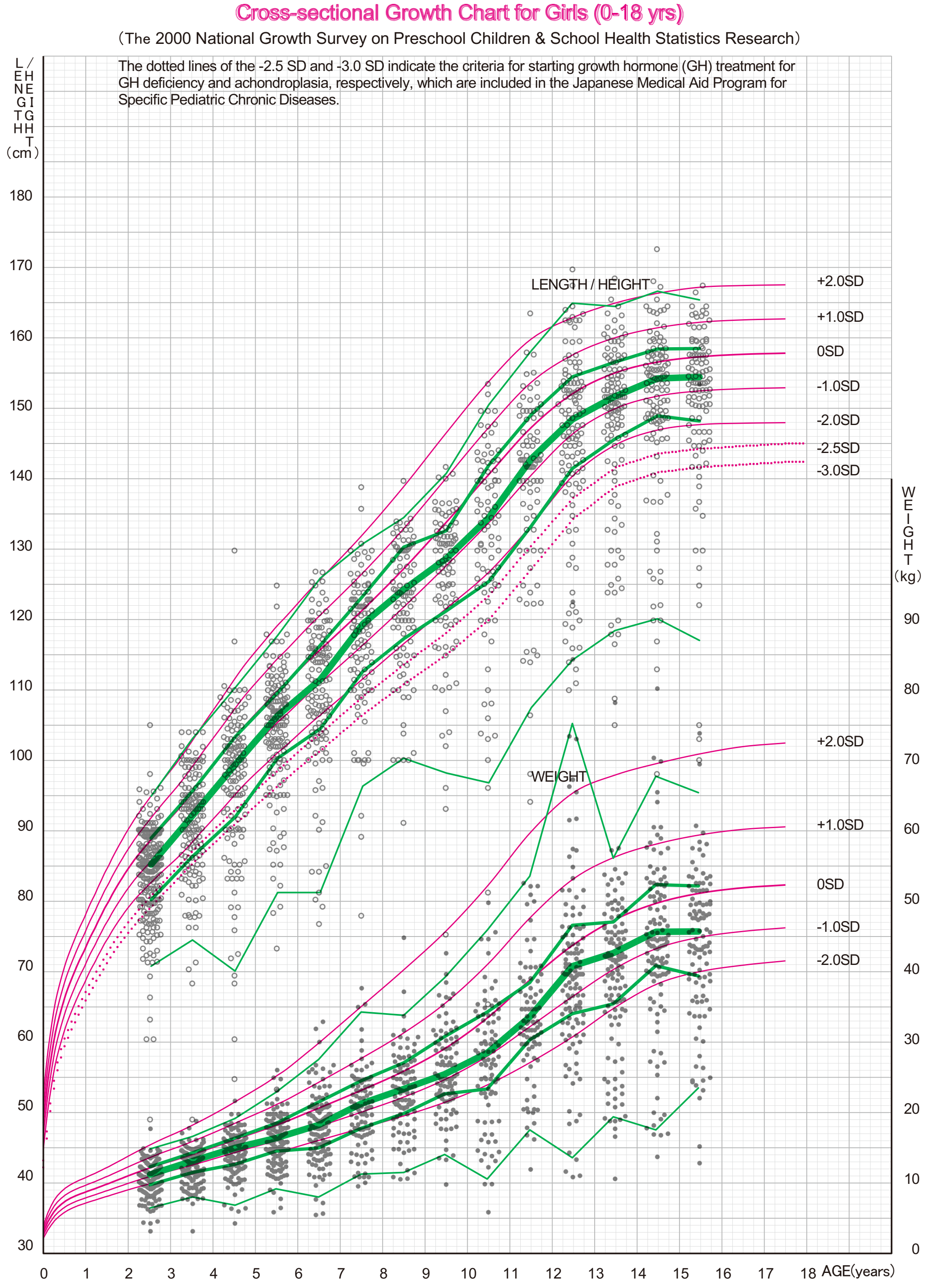

© The Japanese Society for Pediatric Endocrinology
Source: T Isojima, N Kato, Y Ito, S Kanzaki, M Murata, Clin Pediatr Endocrinol 25:71-76, 2016

Supplementary material 2.

Crude data plots with percentile distribution of height (red lines) and bodyweight (green lines) overdrawn on the growth charts for Japanese children (reference #2). Each line was drawn by connecting percentile data of each age with straight line. Hollow and solid circles indicate height and bodyweight of each subjects, respectively. The 1st (fine), 2nd (middle), 3rd (bold), 4th (middle), and 5th (fine) lines from the top indicate 97.5 percentile, 75.0 percentile, 50 percentile, 25 percentile and 2.5 percentile, respectively. Blue and pink lines indicate standard deviation distribution of male and female Japanese reference pediatric population, respectively, calculated by LMS method (reference #2). The original growth charts are available at online by the Japanese Society for Pediatric Endocrinology (JSPE; http://jspe.umin.jp/medical/chart_dl.html, Accessed on April 2021. The charts were reproduced with official permission of the Japanese Society for Pediatric Endocrinology from Isojima et al. Growth standard charts for Japanese children with mean and standard deviation (SD) values based on the year 2000 national survey. Clin Pediatr Endocrinol 25: 71-76, 2016 [reference #2].)

Supplementary material 3. Average values of bodyweight and height for each age

Age	Male									Female								
	n	Weight (kg)				Height (cm)				n	Weight (kg)				Height (cm)			
		Japanese national mean*	Upper 95%CI of ICU mean	ICU Mean	Lower 95%CI of ICU mean	Japanese national mean*	Upper 95%CI of ICU mean	ICU Mean	Lower 95%CI of ICU mean		Japanese national mean*	Upper 95%CI of ICU mean	ICU Mean	Lower 95%CI of ICU mean	Japanese national mean*	Upper 95%CI of ICU mean	ICU Mean	Lower 95%CI of ICU mean
0 month	223	3.00	3.124	3.037	2.950	49.0	49.46	49.00	48.54	163	2.95	2.886	2.806	2.725	48.5	48.17	47.67	47.16
1 month	124		4.165	4.021	3.877		53.11	52.48	51.84	121		3.709	3.571	3.433		51.12	50.42	49.72
2 months	104		4.899	4.713	4.528		55.85	54.96	54.08	88		4.677	4.494	4.311		55.41	54.50	53.59
3 months	97	6.31	5.471	5.246	5.021	61.5	59.02	58.16	57.30	92	5.86	5.228	5.022	4.816	60.1	58.50	57.59	56.67
4 months	76		6.198	5.911	5.623		62.15	61.21	60.26	73		5.641	5.359	5.077		59.78	58.78	57.78
5 months	78		6.293	6.000	5.708		62.28	61.04	59.80	70		6.062	5.753	5.444		61.68	60.73	59.78
6 months	77	7.93	6.907	6.563	6.219	67.7	64.46	62.98	61.50	60	7.32	6.959	6.585	6.210	66.2	64.33	63.02	61.72
7 months	54		7.652	7.306	6.959		67.62	66.26	64.90	52		7.040	6.654	6.267		65.57	64.15	62.72
8 months	59		7.747	7.233	6.719		67.7	65.91	64.11	65		6.967	6.640	6.313		65.59	64.36	63.13
9 months	56	8.80	7.806	7.367	6.928	71.6	68.29	66.89	65.50	42	8.14	7.542	7.099	6.656	70.2	69.66	67.63	65.59
10 months	53		8.584	8.146	7.708		71.53	70.34	69.16	36		7.218	6.810	6.402		67.37	66.03	64.69
11 months	51		8.770	8.434	8.098		71.36	69.86	68.37	48		8.226	7.856	7.485		70.86	69.31	67.77
12 months	67	9.38	8.685	8.338	7.991	74.8	72.81	71.28	69.75	45	8.72	8.451	8.014	7.578	73.5	71.97	70.47	68.98

13 months	61		8.849	8.428	8.008		73.47	71.70	69.93	40		8.577	8.150	7.723		75.64	72.98	70.33
14 months	51		9.155	8.821	8.488		74.54	72.95	71.35	37		8.678	8.019	7.361		72.80	70.34	67.88
15 months	46	9.91	9.189	8.710	8.231	77.8	74.63	73.31	71.99	32	9.26	8.784	8.355	7.925	76.6	73.22	71.59	69.96
16 months	40		9.509	8.992	8.476		77.02	75.34	73.65	32		9.207	8.718	8.229		76.05	74.57	73.08
17 months	27		9.850	9.296	8.741		77.57	75.81	74.04	29		8.585	7.906	7.227		73.89	71.37	68.85
18 months	33	10.4	10.23	9.769	9.309	80.7	79.14	78.04	76.94	39	9.82	9.616	9.058	8.499	79.5	77.51	75.42	73.33
19 months	35		10.18	9.751	9.323		79.47	77.89	76.32	31		9.711	9.082	8.454		78.94	76.48	74.03
20 months	35		10.25	9.705	9.160		79.76	77.42	75.07	31		9.895	9.256	8.616		77.69	75.49	73.28
21 months	35	11.0	10.54	9.851	9.164	83.4	79.86	77.57	75.29	24	10.4	9.505	8.725	7.946	82.2	79.52	76.9	74.28
22 months	32		10.76	10.22	9.675		82.13	80.53	78.93	33		9.945	9.362	8.779		80.13	76.84	73.54
23 months	35		11.11	10.61	10.12		83.16	81.36	79.56	27		10.16	9.466	8.770		80.57	78.15	75.72
2 years	281	12.5	11.38	11.11	10.85	89.7	85.18	84.46	83.73	234	12.1	11.11	10.84	10.57	88.4	84.65	83.77	82.89
3 years	207	14.5	13.81	13.49	13.17	97.1	93.5	92.55	91.59	147	14.0	12.95	12.56	12.16	95.4	91.90	90.59	89.28
4 years	177	16.5	15.43	14.95	14.48	103.6	99.83	98.52	97.21	134	16.1	14.84	14.34	13.84	103.2	98.45	96.6	94.75
5 years	182	18.5	17.57	16.95	16.33	110.1	106	104.4	102.8	149	18.2	16.61	16.06	15.51	109.7	105.3	103.8	102.2
6 years	155	20.9	19.94	19.16	18.37	116.4	112.3	110.8	109.3	122	20.6	18.93	18.05	17.16	115.5	111.4	109.5	107.6
7 years	136	23.5	21.55	20.65	19.75	122.4	118.3	116.4	114.6	103	23.2	21.78	20.71	19.64	121.2	119.0	117.0	115.1
8 years	105	26.4	26.03	24.73	23.43	127.8	125.4	123.1	120.7	97	25.9	24.18	22.99	21.80	127.2	123.9	122.0	120.1
9 years	90	29.6	30.06	28.54	27.02	133.1	132.7	130.5	128.4	94	29.2	27.22	25.95	24.67	133.8	127.8	125.5	123.2
10 years	100	33.4	32.68	30.98	29.27	138.8	137.6	135.3	133.0	86	33.6	29.32	27.41	25.51	140.6	134.0	130.6	127.3
11 years	92	38.1	35.21	33.11	31.02	145.4	140.5	138.0	135.5	85	39.0	35.05	33.18	31.30	147.2	142.0	139.1	136.2

12 years	132	43.6	40.34	38.27	36.21	153.1	148.6	146.3	144.0	120	43.8	42.81	40.38	37.95	152.1	148.2	145.8	143.4
13 years	112	49.0	48.39	45.59	42.79	160.5	157.3	155.2	153.1	122	47.5	41.37	39.44	37.51	155.1	150.3	148.0	145.7
14 years	135	54.0	50.06	47.72	45.38	165.6	162.0	159.4	156.9	118	49.8	46.21	44.02	41.83	156.6	152.7	150.4	148.1
15 years	134	57.5	51.93	49.34	46.76	168.6	163.5	160.7	158.0	108	51.2	47.20	44.69	42.18	157.3	153.7	151.4	149.1

* The value the “Japanese national mean” is derived from the published data on growth standard charts for Japanese children [ref.2].

After 2 years, the value used the half-a-year point data of each age (e.g. Published data of 5.5 year-old for 5 year-old ICU children).
